# Distinct Presentations of Hernia of Umbilical Cord

**DOI:** 10.21699/jns.v5i4.400

**Published:** 2016-10-10

**Authors:** Bilal Mirza, Waqas Ali

**Affiliations:** 1 Department of Pediatric Surgery, Children Hospital Faisalabad, Pakistan; 2 Department of Pediatric Surgery, National Institute of Child Health, Karachi, Pakistan

**Keywords:** Hernia of umbilical cord, Intestinal obstruction, Evisceration of intestine, Prolapsed Patent vitellointestinal duct, Congenital short gut, In-utero volvulus, Intestinal atresia

## Abstract

Hernia of umbilical cord is a well-known entity which presents with herniation of small bowel into the proximal part of umbilical cord. It has very good prognosis after surgical repair. Occasionally, it can have distinct presentations and varied malformations at the umbilicus which have bearing on the course of treatment and final outcome. Herein, we describe various presentations and malformations associated with hernia of umbilical cord. Embryological extrapolation is attempted for the malformations at umbilicus.

**Introduction:**

In past few years, we encountered various perplexing malformations at umbilicus associated with hernia of umbilical cord (HUC), few of these were already reported by us. [1-3] Given our experience of dealing with these different bowel malformations associated with HUC, an explication to the formation of these malformations during intrauterine life is presented here. We have also conducted a perusal of literature to support our assumptions. Various presentations are also summarized for better understanding (Table 1). 

**Figure:**
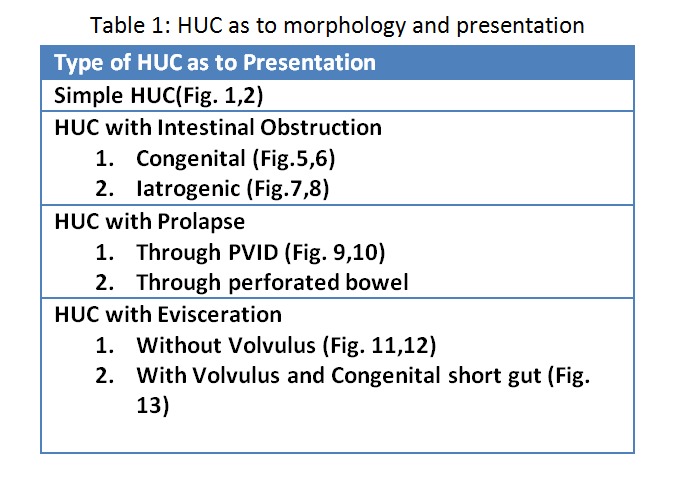
Table 1: HUC as to morphology and presentation

**Embryology:**

Normally, the bowel herniates physiologically into the developing umbilical cord during 5th-6th week of gestation and returns completely back to the abdominal cavity by 12th week of gestation. [4]During this period, the rotation and fixation of bowel takes place. This journey is safe in majority; however, a number of misadventures can occur during this period.[5] The most common malady encountered on account of disruption of normal return of bowel to the abdomen is HUC. Failed closure of umbilical ring or failure of complete return of physiologically herniated bowel is considered the possible mechanisms of formation of HUC.[5] Nevertheless, there are various other events during physiological herniation that result in various malformations at the umbilicus associated with HUC. These are described in the next sections.

**Simple HUC:**

It is the most common variety of HUC and presents with a small swelling at the base of umbilical cord in otherwise normal and asymptomatic neonate. Mainly a small loop of mid ileum is the usual content of HUC but rarely remnants of vitellointestinal tract can be found such as Meckel's diverticulum (Fig. 1). [6] Other contents may be terminal ileum, cecum, (Fig. 2), ascending colon, or entire small bowel even extending till transverse colon, though the size of HUC in that case would be much larger. Rarely entire liver or part of it or accessory lobe of liver, and gallbladder are also reported as contents of HUC.[7-10]The size of herniated contents may range from as small as 2cm, accommodating a small part of small bowel, to more than 10cms harboring many loops of small bowel which may often mimic as omphalocele (Fig. 3).[1] Simple HUC can be treated surgically with a very good prognosis. A gentle squeeze during initial few hours of birth usually leads to reduction of the contents followed by umbilicoplasty (Fig. 4). 

**Figure:**
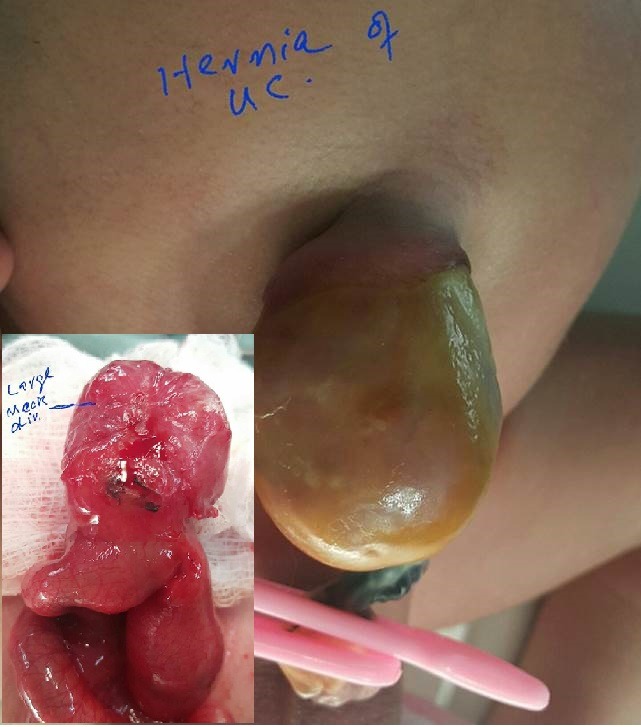
Figure 1: Small hernia of umbilical cord. Inset shows Meckel's diverticulum as a content of HUC. [Courtesy of Dr. Asif Qureshi, Associate Professor of Pediatric Surgery, at Jinnah Hospital Lahore]

**Figure:**
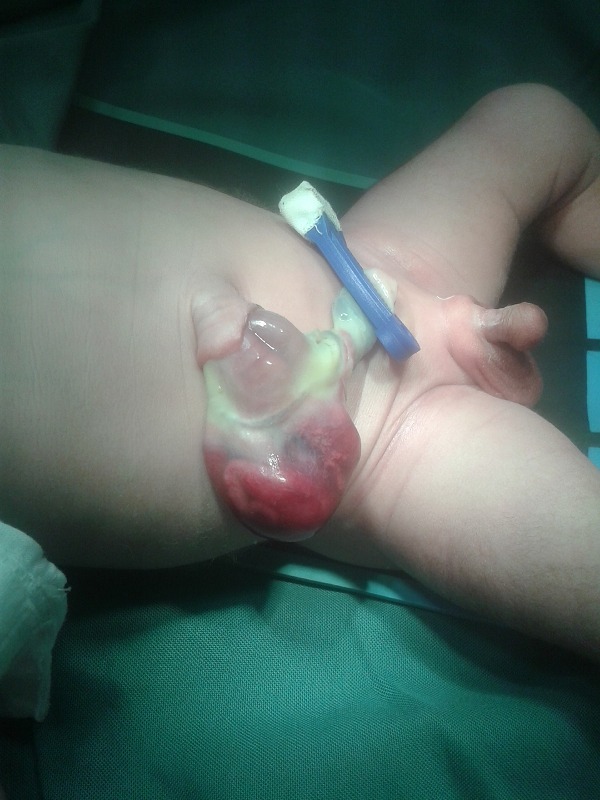
Figure 2: HUC having terminal ileum, cecum, and appendix as contents.

**Figure:**
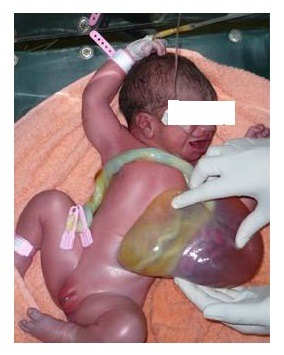
Figure 3: Large HUC containing many loops of bowel mimicking as giant omphalocele. The child was cyanotic and had cardiac anomalies.

**Figure:**
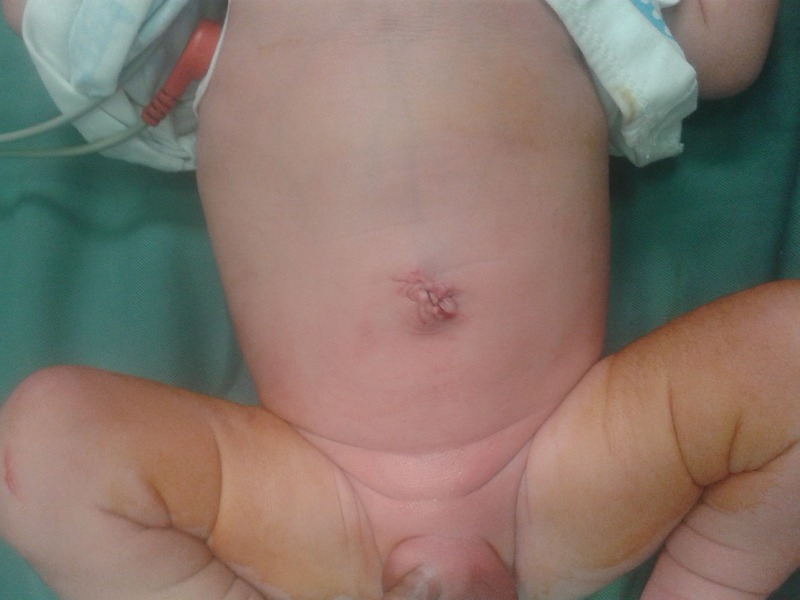
Figure 4: Umbilicoplasty after reduction of HUC.

**HUC with Intestinal Obstruction:**

As the name indicates, this type of HUC presents with features of intestinal obstruction which may be congenital or acquired/iatrogenic in etiology. The congenital intestinal obstruction associated with HUC is mainly due to intestinal atresia.[2,3,11]In-utero vascular accidents in the form of volvulus or compression from the umbilical ring are considered the causes of intestinal atresia.[2,3,11]A case of in-utero small bowel perforation proximal to ileal atresia, within the HUC, is also reported in literature.[11]

HUC associated with intestinal atresia may occasionally have very short length bowel.[3] A vascular event secondary to volvulus of herniated midgut during physiological herniation or a narrow umbilical ring may occlude mesenteric blood supply resulting in complete resorption of the herniated midgut are the possible phenomenon behind it. We have reported a case of HUC associated with complete loss of mid gut. [3] On removing umbilical cord there were two blind loops of bowel being herniated from the umbilical ring. Further exploration revealed less than 10cm of proximal jejunum and almost same length of distal sigmoid colon; rest of the entire midgut has been lost in-utero (Fig. 5). 

**Figure:**
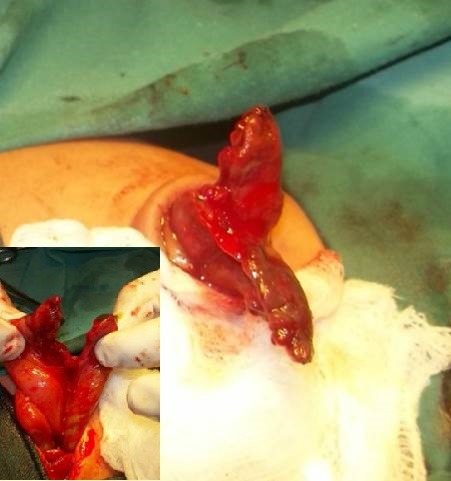
Figure 5: HUC with congenital loss of midgut. Inset shows rest over few inches of proximal jejunum and distal sigmoid colon. The rest of entire gut was lost. [3]

Occasionally a relatively dilated and meconium filled loop of small bowel may get entrapped in the umbilical cord while returning to the abdomen. Due to continued pressure of the closing umbilical ring, it may get sequestered from the rest of the bowel and presents as content of HUC. It is associated with entry and exit level atresias at the level of umbilical ring (Fig. 6). Twist of the herniated loop could be the other plausible explanation of the event leading to isolation of the entrapped bowel segment.

**Figure:**
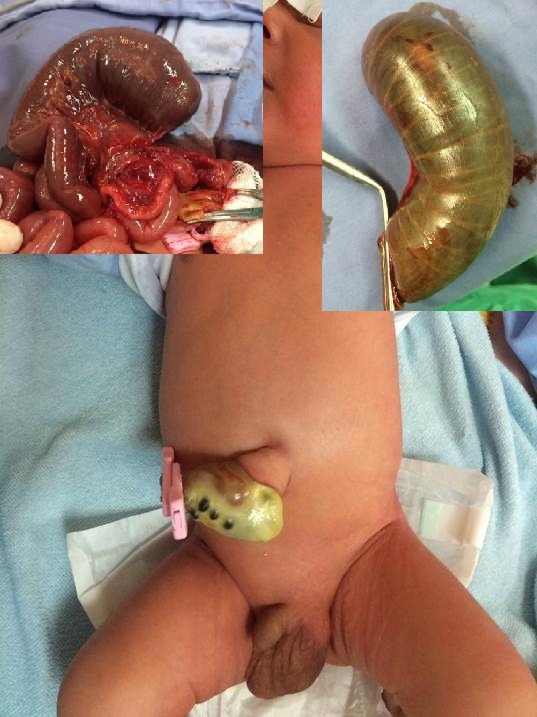
Figure 6: The patient presented with HUC on exploration a sequestered meconium filled loop was found as HUC content (left inset). The other inset shows associated distal ileal atresia. [Courtesy of Professor Naeem Zafar Khan of KRL Hospital and National Institute of Handicapped Islamabad, Pakistan]

We have encountered few cases of HUC presented with intestinal obstruction without any atresia. We believe that presence of only small part of small bowel in HUC does not lead to intestinal obstruction. Occasionally, the presence of terminal ileum, cecum and ascending colon (Fig.2) in HUC may produce obstructive symptoms. The reasons may be speculated as compression on the herniated bowel, small size of umbilical ring, and kinking of the ileocecal valve. In such cases more adhesions of the herniated contents with the sac, are observed during surgery. This is not always true as Gajdhar et al [10] reported case of giant HUC where entire small bowel from proximal jejunum to the ascending colon was herniated to the HUC but patient did not develop intestinal obstruction. On the other hand, Ali et al[1] reported a case of giant HUC harboring many bowel loops, few of which were compromised thus mimicked as herniation of liver tissue. They initially thought it omphalocele but later on patient deteriorated clinically and exploration divulged it as HUC containing many loops of small bowel; few loops were compromised and needed resection-anastomosis of the bowel. 

Iatrogenic injury to the herniated contents makes handful number of cases of HUC with intestinal obstruction. It is usually caused by accidental ligation or clamping of the HUC (Fig.7,8). [12,13] Untrained birth attendants or lack of awareness about the entity is the main cause of application of ligature/clamp over the HUC resulting in intestinal obstruction. The ligature or clamp can cut through the intestinal contents leading to meconium discharge at the level of injury.[12,13] 

**Figure:**
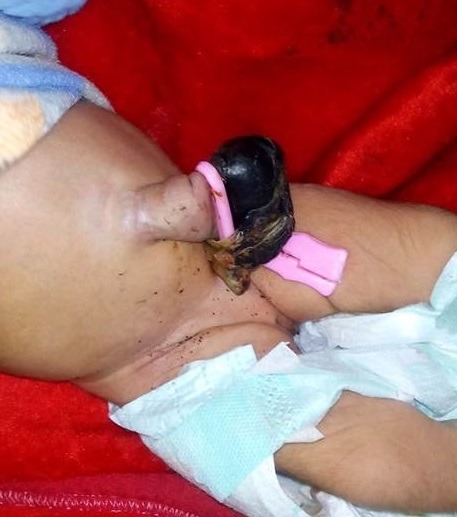
Figure 7: Accidental application of clamp over the HUC.

**Figure:**
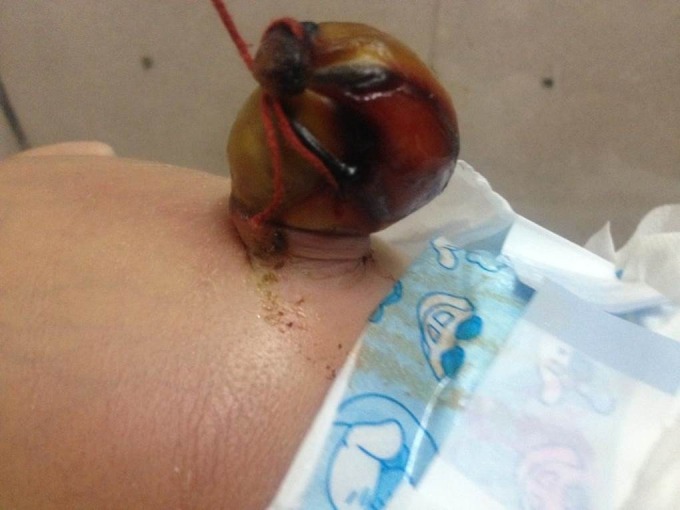
Figure 8: HUC hardly escaped injury by thread ligature by a birth attendant in village setting.

All these cases need investigations in the form of abdominal radiograph, and ultrasound to see level and nature of intestinal obstruction although it is overt clinically. More extensive surgery is required needing resection of bowel and restoration of continuity of bowel or formation of temporary stoma. The outcome is good but occasionally patients may have a stormy outcome and may die especially those with complete loss of mid gut or who developed septicemia secondarily.[3] 

**HUC with Mucosal Prolapse:**

This is a special variety of HUC which presents with mucosal prolapse from the sac of HUC. The prolapse in majority is secondary to the presence of patent vitellointestinal duct (PVID) (Fig. 9, 10). Occasionally, an in-utero perforation of herniated small or large bowel during physiological herniation phase may also lead to prolapse of mucosa from the sac of HUC.[14]

**Figure:**
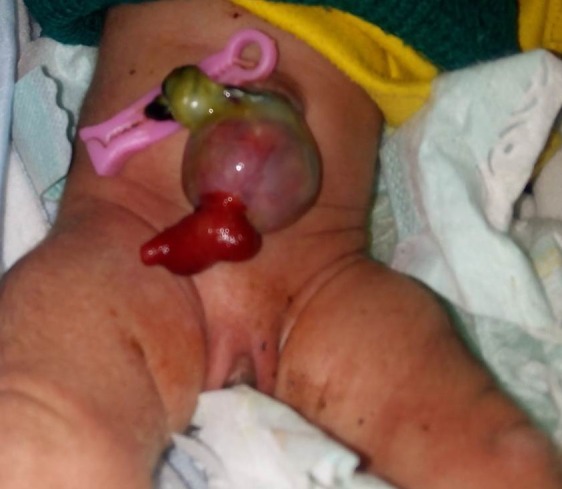
Figure 9: HUC with prolapse of PVID.

**Figure:**
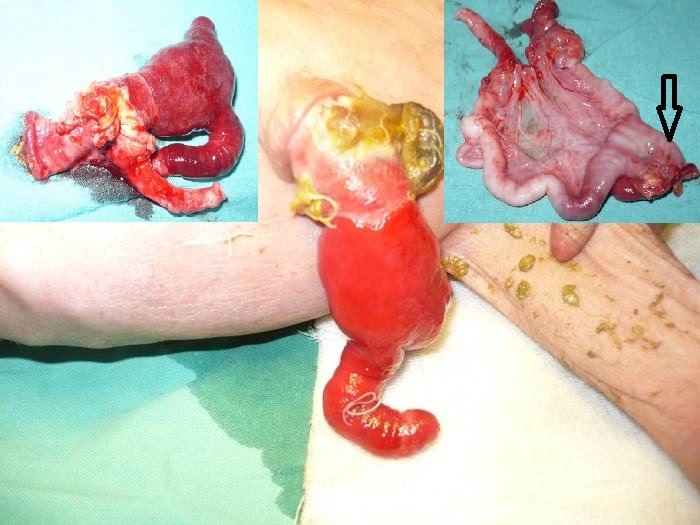
Figure 10: HUC with prolapsed PVID. At operation the prolapsed part could not be reduced back thus necessitating resection and anastomosis. The prolapsed resected gut when reduced forcefully revealed significant length of gut. Arrow shows the PVID.

Embryologically, developing intestines are communicated with yolk sac through a Vitellointestinal tract at the summit of mid gut. This connection is lost during 5-7th week of gestation when the midgut is physiologically herniated in the umbilical cord coelom. When there is failure of resorption of this tract completely, a PVID results. It can be speculated that the PVID, which has one free mucosal lined end, may get adhered to the umbilical cord and causes its localized rupture or resorption; the mucosa may then be prolapsed through the wall of HUC as a result of peristalsis as normally happens in case of enterostomy. Similar events might be presumed in case of in-utero bowel perforation within the HUC. Mucosal prolapse is not always part of this event.[14] The prolapsed mucosa hangs into the amniotic cavity and excretes meconium leading to meconium staining of the fetus and meconium aspiration syndrome.[11]

These patients on antenatal scan may mimic as gastroschisis owing to direct floating of bowel mucosa in the amniotic cavity.[11] At birth, these patients present with HUC with a prolapsed mucosa from sac of HUC and pass meconium from the prolapsed mucosa. These patients may exclusively pass stool from prolapsed mucosa in case of distal obstruction such as associated distal atresia but can occasionally pass meconium per-anally in case of PVID associated prolapse. The surgical approach is by trans-umbilical or semilunar infra-umbilical incision. The prolapsed bowel is reduced back and PVID can be resected with restoration of the normal bowel continuity by anastomosis. In case of massive prolapse and edematous mucosa not able to be reduced back, a more extensive resection and anastomosis is performed to restore the continuity (Fig. 10). 

**HUC with Bowel Evisceration:**

This is extremely rare variety of HUC.[2]Although these patients present with typical features of neonatal intestinal obstruction, but owing to presence of bowel evisceration are grouped in this category. During physiological herniation of bowel, the umbilical coelom may get ruptured leading to evisceration of herniated bowel. The reason of rupture of umbilical cord is not known as yet. It may happen secondary to bowel dilatation with associated intestinal atresia or there might be some vascular accident such as rupture of umbilical vessels which may become fatal to the fetus. Haas et al [5] described such an occurrence in a fetus which was on follow-up for HUC and developed in-utero evisceration of bowel through the umbilical cord. This led to demise of the fetus. On autopsy, a wall of umbilical cord was ruptured along with rupture of umbilical vessels which caused to fatality. 

After evisceration, the bowel loops float freely in the amniotic cavity as happens in gastroschisis. If no further devastating events take place, these patients are born with eviscerated bowel from one side of umbilical cord. The bowel and mesentery look edematous and is covered by a thick peel of fibrin; the entity usually mimics as gastroschisis,[2] on initial inspection (Fig. 11). Careful and detailed inspection will differentiate it from gastroschisis owing to lack of an abdominal wall defect to the right side of umbilical cord. It is usually associated with entry/exit level atresias.[2]Occasionally the eviscerated loops may mat together and present as a discoid mass of bowel over the umbilicus (Fig. 12). 

**Figure:**
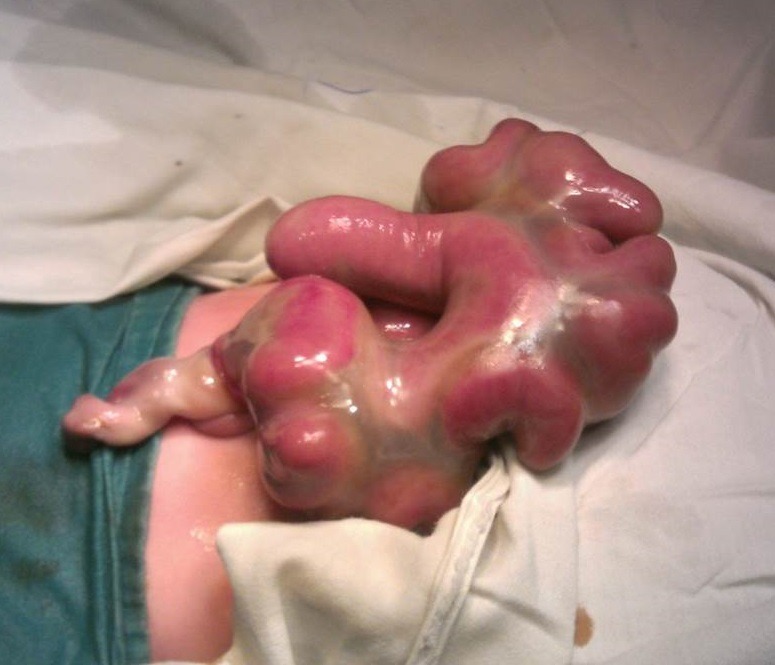
Figure 11: HUC with evisceration of bowel from the umbilical cord mimicking as gastroschisis on initial inspection.[2]

**Figure:**
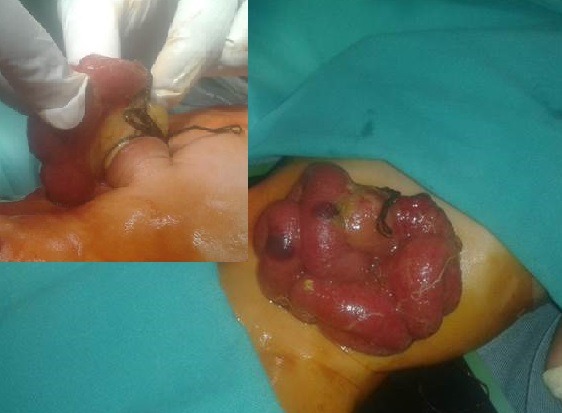
Figure 12: HUC with evisceration of bowel. The bowel is matted to form a discoid mass of gut over the umbilicus. Inset shows application of the ligature by an untrained birth attendant in village setting.

It is likely that the freely floating eviscerated bowel in the amniotic cavity may get twisted leading to loss of entire mid gut. These patients present with a small bowel mass extruded out from one side of the umbilicus (Fig. 13). On exploration, there is extremely short length of bowel; merely 5-10cms of proximal jejunum and 5-10cm of distal colon, rest of entire bowel is lost due to in-utero volvulus (Fig. 13). 

**Figure:**
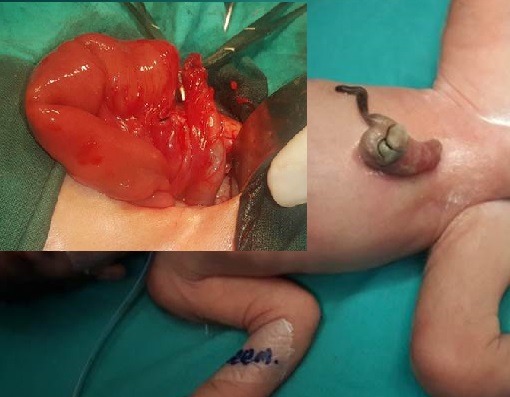
Figure 13: HUC with evisceration of bowel mass from umbilical cord. Inset shows rest over few inches of proximal jejunum and distal sigmoid colon. The rest of entire gut has been lost in a presumable in-utero volvulus of the eviscerated bowel from the umbilical cord. [Courtesy of Dr. Nabi Bux, Dept. of Pediatric Surgery, The Children's Hospital and the Institute of Child Health, Lahore]

Abdominal radiography shows 2-3 air fluid levels and ultrasound abdomen also depicts few dilated bowel loops. Exploration is mandatory to identify total length of bowel. In case where eviscerated bowel is healthy and not associated with volvulus or midgut loss, the outcome is good.[2] The associated entry/exit atresias are managed primarily. We have reported such a case where proximal atresia was primarily managed by end to end anastomosis and at the level of distal colonic stenosis, an enterostomy was formed which worked well in our case.[2] The outcome is very poor in case of complete loss of mid gut leaving few centimeters of proximal jejunum and distal rectosigmoid colon (Fig. 13).These patients can be initially managed on TPN and later on gut transplantation may be an option for these children although not available In Pakistan as yet.

**Associated anomalies:**

Hernia of umbilical cord has been associated with very few malformations especially of alimentary tract. We have reviewed the literature regarding associated malformations and presented our findings in Table 2. 

**Figure:**
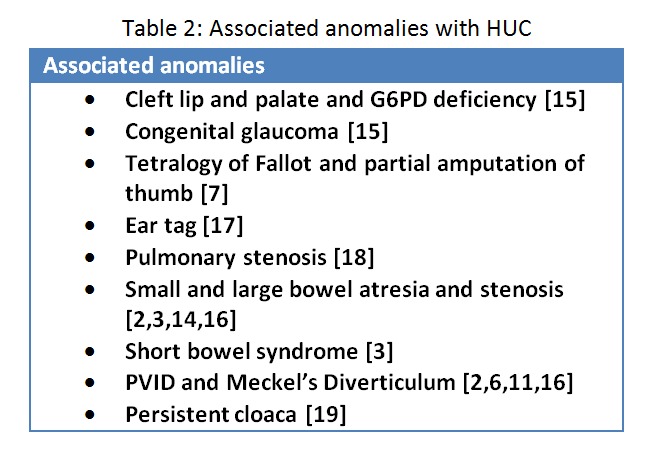
Table 2: Associated anomalies with HUC

**Summary of Embryological Events:**

A summary of the possible embryological events and its correlation with the clinical presentation and the malformations at the umbilicus associated with HUC is presented as follows: physiological herniation of mid gut occurs into the developing umbilical cord during 5-12th week of gestation. Simple hernia of umbilical cord can result owing to failure of bowel to return back completely. The bowel during physiological herniation may get volvulus inside the umbilical coelom resulting in HUC with loss of entire midgut. Occasionally the bowel, during physiological herniation may get evisceration through a rupture of umbilical cord. The freely floating bowel may mimic gastroschisis at birth. If the freely floating bowel in the amniotic cavity gets a twist, it may lead to extensive bowel loss and extremely short length bowel. Lastly a meconium filled part of bowel may entrap in the umbilical cord and isolate owing to a very narrow umbilical defect.

## Footnotes

**Source of Support:** Nil

**Conflict of Interest:** None
